# Epidemiology, Treatment and Prognosis Analysis of Small Cell Breast Carcinoma: A Population-Based Study

**DOI:** 10.3389/fendo.2022.802339

**Published:** 2022-04-04

**Authors:** Jiahao Zhu, Gang Wu, Yutian Zhao, Bo Yang, Qingqing Chen, Jianwei Jiang, You Meng, Shengjun Ji, Ke Gu

**Affiliations:** ^1^ Department of Radiotherapy and Oncology, The Affiliated Hospital of Jiangnan University, Wuxi, China; ^2^ Department of Radiotherapy and Oncology, The Affiliated Suzhou Hospital of Nanjing Medical University, Gusu School, Nanjing Medical University, Suzhou, China; ^3^ Department of Breast Surgery, The Affiliated Suzhou Hospital of Nanjing Medical University, Gusu School, Nanjing Medical University, Suzhou, China

**Keywords:** breast, small cell carcinoma, epidemiology, prognosis, disease-specific survival, overall survival

## Abstract

**Background:**

Primary small cell breast carcinoma (SCBC) is an uncommon malignancy with highly invasive behavior. The aim of this study was to find out more about the incidence, clinicopathologic characteristics and identify potential prognostic factors of SCBC.

**Methods:**

Data of patients with primary diagnosis of SCBC between 1975 and 2018 were extracted from the Surveillance, Epidemiology, and End Results (SEER) database. The incidence after adjustment for age and percentage change per year in incidence were calculated. Disease-specific survival (DSS) and overall survival (OS) were analyzed among these SCBC patients identified from the SEER database. The whole cohorts were randomized into training and validation cohorts as ratio of 7: 3. Cox regression analysis was performed to determine predictors of survival with the training cohorts. Predictive models were constructed with training cohorts, and nomogram validation was performed using receiver operating characteristic curves, concordance indices and calibration curves in both training and validation cohorts.

**Results:**

323 SCBC patients were enrolled finally during the research period. The overall incidence after adjustment for age between 1990 and 2018 was 0.14 per million per year, and the prevalence of the incidence has plateaued. Most of these tumors were poorly differentiated or undifferentiated. The most prevalent presenting stage was Stage II. Patients identified in this study were randomly divided into training (n = 226) and testing (n = 97) cohorts. Multivariate Cox proportional hazards model showed that chemotherapy, surgery and stage were important predictors of DSS and OS.

**Conclusion:**

SCBC is considered an infrequent breast neoplasm with aggressive characteristics. Tumor stage is associated with poor prognosis. Combination of surgery and chemotherapy is the main treatment for SCBC.

## Introduction

Primary small cell breast carcinoma (SCBC) is an uncommon neoplasm that makes up less than 1% of all invasive breast cancer cases and approximately 7% of all extrapulmonary small cell carcinomas (1, 2). SCBC, a subtype of neuroendocrine neoplasm, was first described by Wade et al. in 1983, and diagnostic criteria were first proposed by Sapino et al. in 2000 (3, 4). Because of the rarity of instances, an agreement on the nomenclature and diagnostic criteria of SCBC could not be reached for a long period. Recently, a new classification system arisen by the World Health Organization expert panel defined SCBC as a neuroendocrine carcinoma with poor differentiation (1). A study published in 2021 demonstrated that disease-specific survival (DSS) and overall survival (OS) of neuroendocrine neoplasm was significantly worse than invasive ductal carcinoma of no special type (All *P* < 0.001). However, further analyse of SCBC was not conducted in this study (5).

In terms of treatment, the standardized therapy protocol for SCBC is largely undefined. Given the similar histologic and morphologic features with small cell lung cancer (SCLC), the current clinical management of SCBC is mostly extrapolated from the therapeutic strategies of SCLC, mainly combining surgery, chemotherapy and radiotherapy. The main chemotherapy schedules used include etoposide and platinum agents, even anthracycline and taxane (6). Morever, A case of SCBC patient treated with regimen of doxorubicin and cyclophosphamide then followed by carboplatin and etoposide achieved favourable therapeutic effect (7)[Append 21]. The administration of adjuvant radiotherapy is given based on the size of tumor and status of lymph node (8). Moreover, endocrine treatment is added when SCBC expresses the relative hormone receptors (9, 10). About 75% of SCBC patients were detected with TP53 mutations and 33% cases detected with PIK3CA mutations, which means TP53 and PIK3CA could serve as the potential therapeutic targets (11). Because of the limited number of prospective studies and large sample investigations for consolidating the medical evidence of SCBC, the standard for a definitive and preferable management strategy varies among different medical institutions or clinicians.

In our study, we gathered a sizable sample of SCBC patients and extracted clinical information from the Surveillance, Epidemiology and End Results (SEER) database for analysis. We investigated the incidence, tumor characteristics and outcomes of SCBC and explored optimal treatments and potential prognostic factors for SCBC.

## Materials and Methods

### Patients Selection

**Figure 1:**
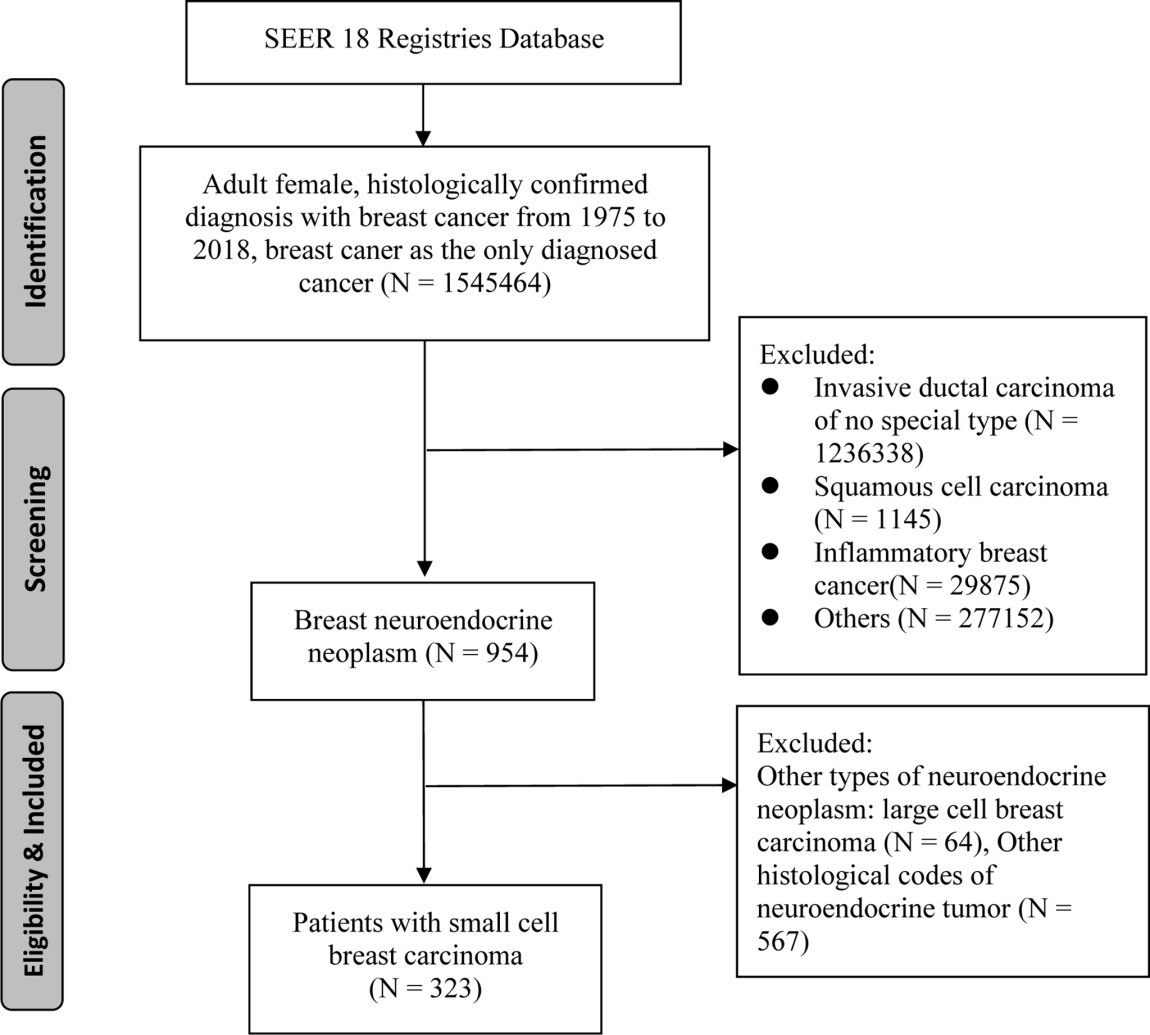
Flow diagram of patients enrollment in this study.

The SEER 18 Registries data set was utilized to identify individuals who were first diagnosed with SCBC from 1975 to 2018 (12). Patients with SCBC were identified in the SEER database using topographical and histology codes from the International Classification of Diseases (ICD-O-3). C50.0 to C50.6, C50.8, and C50.9 were the topographical codes utilized in this investigation. Cases of small cell carcinoma was identified using the ICD-O-3 histological codes 8041, 8042, 8043, 8044, and 8045. Clinical, pathological and survival information was collected by using SEER*Stat version 8.3.6. Patients who met following criteria were recruited in to our study: 1) female patient with age more than 18 years; 2) The diagnosis of SCBC was confirmed by pathology; 3) SCBC was the primary cancer and no other primary cancers; 4) complete survival duration and cause of death. Finally, we identified a total of 323 patients for this study cohort (**Figure 1**).

Primary cohort with 323 patients met enrollment criteria was further divided into training cohort (n = 226) and validation cohort (n = 97) randomly as ratio of 7:3. Training cohort with 226 patients were used to construct the nomograms and validation cohort with 97 cases were used for nomograms validation.

### Endpoint Definition

Patients with a first primary diagnosis of SCBC who had complete staging and survival information were chosen for survival analysis. The endpoints of this research were (DSS, which was the interval between the primary diagnosis of SCBC to the death related with SCBC, and OS, which was the interval from the primary diagnosis of SCBC to death or the last visit.

### Epidemiological Analysis

The incidence rates were calculated, and age was adjusted to the 2000 US population, as the number of new occurrences per 1,000,000 person-years. The yearly percent change was calculated using the weighted least squares technique. For incidence trend analysis, the percentage change was assessed by comparison to zero.

### Clinical Characteristic Analysis

In the current study, Grade I or highly differentiated was defined as G1, grade II or moderately differentiated as G2, and grade III or badly differentiated and grade IV or undifferentiated as G3. For all recruited patients, Kaplan–Meier curves were used to predict survival rates, and the log-rank test was used to examine differences in survival distributions across groups. The multivariate Cox model was used to determine independent prognostic variables with training cohort. Then, A nomogram based on the regression coefficients of each element in the multivariate study was used to visualize the prediction model with training cohort. Validation cohort was used for external validation. Survival prediction value of the nomograms was calculated by performing the area under the receiver operating characteristic (ROC) curve (AUC), calculating concordance index (C-index) and conducting calibration curves. Specificity and sensitivity were derived from the areas under the ROC curves (AUCs). The AUC was used to assess the signature’s prediction abilities. The C-index was used to assess the prediction accuracy and discriminating capabilities of each component and the nomogram. To test the nomogram’s calibration, calibration curves (500 bootstrap resamples) were produced.

### Immunohistochemistry

Pathology and immunohistochemistry were conducted with SCBC tissue. CD56 and Syn localization and Ki-67 expression were evaluated with immunohistochemistry. The tumor tissues of one SCBC patient from Jiangnan University’s Affiliated Hospital were sliced in order, then dewaxed and rehydrated in graded alcohols. The slides were stained with immunohistochemistry according to the manufacturer’s directions. Antibodies for the identification of CD56, Syn and Ki-67 protein expression were purchased from Santa Cruz Biotechnology (Santa Cruz, CA, USA). An Aperio pathology workstation (Aperio) was used for quantitative evaluation. The percentage of cells that stained positively was automatically calculated. The immunohistochemistry data and use of tumor tissue for this investigation were approved by the patient and the institutional review board of the Affiliated Hospital of Jiangnan University.

### Statistical Analysis

R software version 3.6.0 and SPSS version 24.0.0 (SPSS, Chicago, IL) were used for statistical analysis. The primary cohort was randomized into training cohort and testing cohort using R software. All tests were two-sided. A P value < 0.1 was defined as the criterion for eliminating variables in the multivariate Cox model, and a P value < 0.05 was considered to be significant for further testing.

## Results

### Incidence

**Figure 2:**
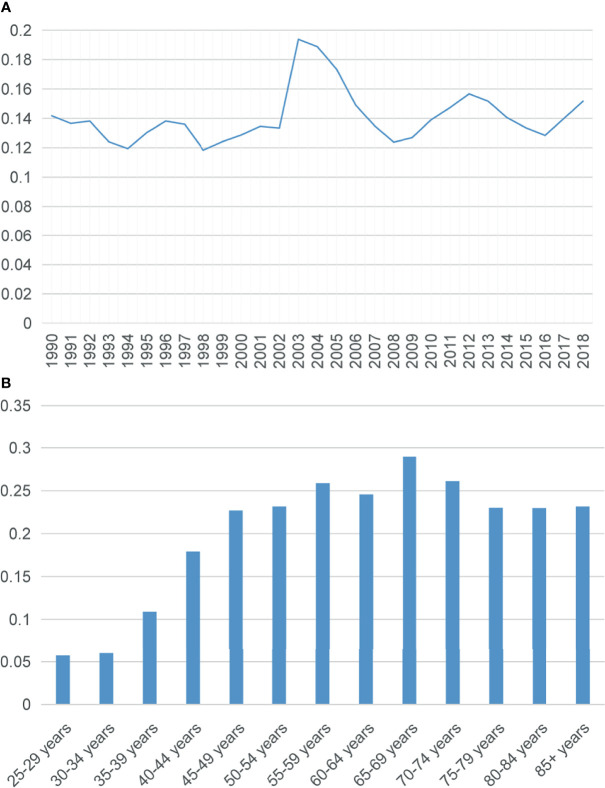
**(A)** Trend in the incidence of small cell carcinoma of the breast from 1990 to 2018. **(B)** Age-wise incidence. All rates were per 1,000,000, and age was adjusted to the 2000 US standard population.

Due to the limitation of population data acquisition, only the incidences of SCBC between 1990 and 2018 were calculated. The overall age-adjusted incidence of SCBC was 0.14 per 1,000,000 per year during this period. The incidence of SCBC reached a peak in 2003 (0.19 per 1,000,000), but the overall prevalence of the incidence has plateaued during the last 28 years (**Figure 2A**). The incidence was the highest in the 65–69 age group (**Figure 2B**).

### Clinical Characteristics and Survival

A total of 323 patients initially diagnosed with SCBC were recruited during the study period. At the time of diagnosis, the median age was 65 years (range: 28–97 years). The most frequent stage among these individuals was stage II and the median tumor size was 3.5 cm. The tumors were mostly poorly differentiated or undifferentiated (133/155). The majority of the patients (94/152) tested negative for estrogen receptor (ER) and progesterone receptor (PR) expression. Of note, human epidermal growth factor receptor 2 (HER-2) status was only reported in patients after 2010 in SEER database, and only one patient (1/67) had positive HER-2 status. Approximately half of these patients (37/67) had triple-negative breast cancer (TNBC). In terms of treatment, most SCBC patients underwent surgery (238/313). Of these 238 patients, 82 received postoperative adjuvant radiotherapy, and 4 received neoadjuvant radiotherapy before surgery. The summary of clinical characteristics is shown in **Table 1**.

**Table 1:** Patient, tumor and treatment characteristics.

Parameter	All patients (Percent)	Training Cohort (Percent)	Validation Cohort (Percent)	*P*
Total number of cases (1975-2018)	323 (100)	226 (100)	97 (100)	
Age at diagnosis				0.453
Median (years)	65	63	66	
Range	28-97	28-97	30-87	
Tumor size				0.685
Median (cm)	3.5	3.3	3.6	
Range	0.3-15.7	0.3-15.5	0.5-15.7	
Race				0.355
White	275 (85.1)	191 (84.5)	84 (86.6)	
Black	38 (11.8)	29 (12.8)	9 (9.3)	
Other	9 (2.8)	6 (2.7)	3 (3.1)	
Unknown	1 (0.3)	0 (0.0)	1 (1.0)	
Laterality				0.794
Right	155 (48.0)	106 (46.9)	49 (50.5)	
Left	160 (49.5)	114 (50.4)	46 (47.4)	
Bilateral	1 (0.3)	1 (0.4)	0 (0.0)	
Unknown	7 (2.2)	5 (2.2)	2 (2.1)	
Tumor location				0.291
Medial/central	62 (19.2)	45 (19.9)	17 (17.5)	
Outer	125 (38.7)	83 (36.7)	42 (43.3)	
Other	50 (15.5)	32 (14.2)	18 (18.6)	
Unknown	86 (26.6)	66 (29.2)	20 (20.6)	
Hormone receptor status				0.016
ER-/PR-	94 (29.1)	63 (27.9)	31 (32.0)	
ER+/PR-	11 (3.4)	5 (2.2)	6 (6.2)	
ER-/PR+	9 (2.8)	5 (2.2)	4 (4.1)	
ER+/PR+	38 (11.8)	21 (9.3)	17 (17.5)	
Unknown	171 (52.9)	132 (58.4)	39 (40.2)	
HER2				0.476
Positive	1 (0.3)	1 (0.4)	0 (0.0)	
Negative	66 (20.4)	49 (21.7)	17 (17.5)	
Unknown	256 (79.3)	176 (77.9)	80 (82.5)	
Differentiation				0.325
Grade 1	6 (1.9)	5 (2.2)	1 (1.0)	
Grade 2	16 (5.0)	10 (4.4)	6 (6.2)	
Grade 3	133 (41.1)	87 (38.5)	46 (47.4)	
Unknown	168 (52.0)	124 (54.9)	44 (45.4)	
T-stage				0.092
1-2	89 (27.6)	56 (24.8)	33 (34.0)	
3-4	30 (9.3)	25 (11.1)	5 (5.2)	
Unknown	204 (63.1)	145 (64.2)	59 (60.8)	
N-stage				0.173
N0	81 (25.1)	50 (22.1)	31 (32.0)	
N+	68 (21.0)	49 (21.7)	19 (19.6)	
Unknown	174 (53.9)	127 (56.2)	47 (48.5)	
Overall staging (AJCC)				0.107
I	35 (10.8)	24 (10.6)	11 (11.3)	
II	56 (17.3)	33 (14.6)	23 (23.7)	
III	32 (9.9)	27 (11.9)	5 (5.2)	
IV	36 (11.1)	23 (10.2)	13 (13.4)	
Unknown	164 (50.9)	119 (52.7)	45 (46.4)	
Surgery				0.253
Yes	238 (73.7)	146 (64.6)	92 (94.8)	
No	75 (23.2)	52 (23.0)	23 (23.7)	
Unknown	10 (3.1)	8 (3.5)	2 (2.1)	
Radiation therapy				0.088
Yes	89 (27.6)	56 (24.8)	33 (34.0)	
No	234 (72.4)	170 (75.2)	64 (66.0)	
Chemotherapy				0.264
Yes	135 (41.8)	99 (43.8)	36 (37.1)	
No/Unknown	188 (58.2)	127 (56.2)	61 (62.9)	
Marital status at diagnosis				0.052
Single	27 (8.4)	14 (6.2)	13 (13.4)	
Married or ever married	282 (87.3)	200 (88.5)	82 (84.5)	
Unknown	14 (4.3)	12 (5.3)	2 (2.1)	

HER2, human epidermal growth factor receptor 2.

**Figure 3:**
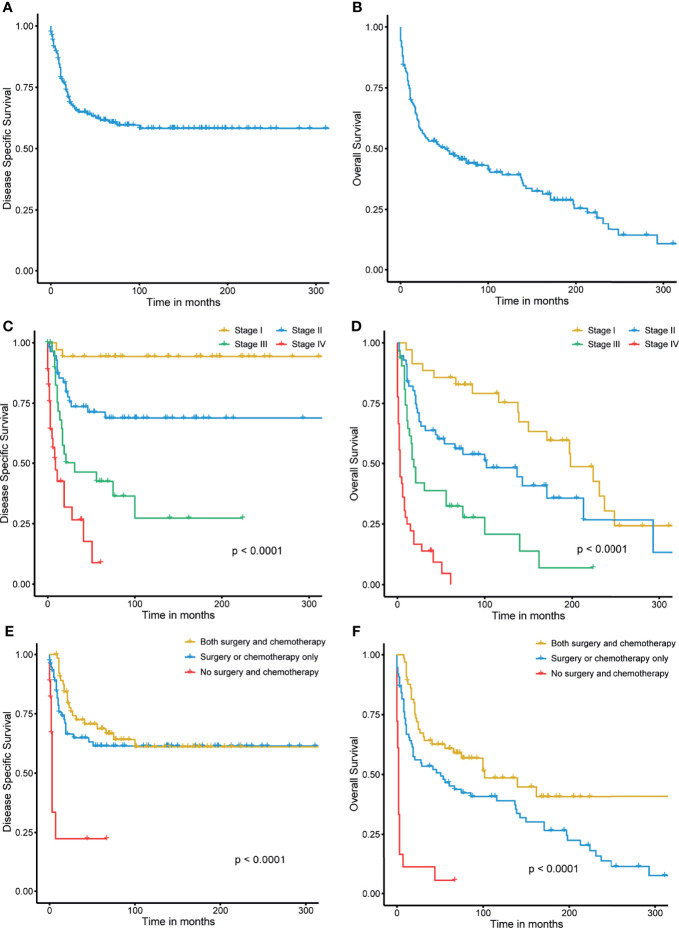
**(A, B)** Disease-specific survival and overall survival of small cell carcinoma of the breast. **(C, D)** Stage-wise disease-specific survival and overall survival of small cell carcinoma of the breast. **(E, F)** Treatment-wise disease-specific survival and overall survival of small cell carcinoma of the breast.

The 3- and 5-year DSS rates were 64.9% and 61.6%, respectively (**Figure 3A**). The 3- and 5-year OS rates were 53.1% and 47.7%, respectively, and the median OS was 50 months (95% confidential interval (CI), 0.432–0.589) (**Figure 3B**). Because some patients had incomplete clinical information, 159 patients with overall stage disease were recruited for further survival analysis. The 3- and 5-year DSS rates for stage I disease were 94.3% and 94.3%, respectively, while those for stage IV disease were 26.6% and 8.8%. The 3- and 5-year OS rates for stage I disease were 88.6% and 85.7%, respectively, while those for stage IV disease were 13.9% and 4.6%, respectively. Significant differences were observed among the different stages in both DSS (P<0.0001) and OS (P<0.0001) (**Figures 3C, D**). Patients who treated with both surgery and chemotherapy had greater DSS and OS rates than those who received just surgery or chemotherapy, or none of the two therapies. (P<0.0001) (**Figures 3E, F**).

**Figure 4:**
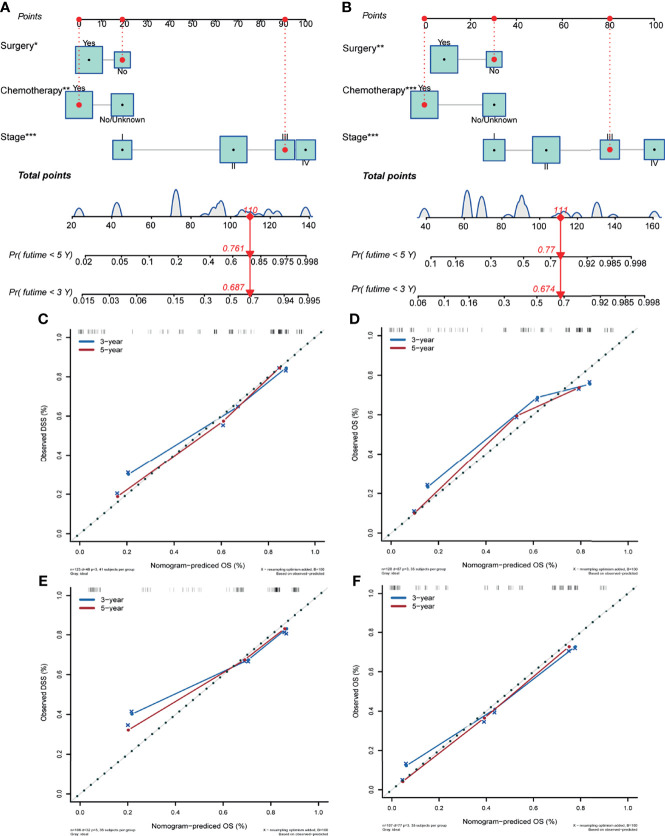
Nomogram predicting the disease-specific survival **(A)** and overall survival **(B)** of patients with initially diagnosed small cell carcinoma of the breast. Calibration plots in the training **(C, D)** and validation **(E, F)** cohorts for 3- and 5-year disease-specific survival and overall survival. Significant differences are defined by *p < 0.05, **p < 0.01, and ***p < 0.001.

Differentiation, tumor location, stage, surgery, radiation therapy and chemotherapy were significantly correlated with poor DSS in the univariate Cox analysis (P<0.10). Age at diagnosis, tumor differentiation, tumor location, stage, surgery, and radiation therapy were related to poor OS (P<0.10). Given the actual clinic practice the risk factor of chemotherapy and these significant variables were enrolled into multivariate analysis. Stage (P = 0.000) (stage II vs. stage I, HR:7.632; 95% CI, 1.105-16.439; P = 0.014) (stage III vs. stage I, HR:26.314; 95% CI, 2.476-68.421; P = 0.000) (stage IV vs. stage I, HR:55.968; 95% CI, 6.678-126.573; P = 0.000), surgery (HR:2.108; 95% CI, 1.063-3.585; P = 0.046), and chemotherapy (HR:3.543; 95% CI, 1.564-6.593; P = 0.003) were the independent variables in DSS, according to multivariate analysis. Stage (P = 0.000) (stage II vs. stage I, HR:2.896; 95% CI, 1.061-6.386; P = 0.019) (stage III vs. stage I, HR:7.472; 95% CI, 2.659-14.351; P = 0.000) (stage IV vs. stage I, HR:21.876; 95% CI, 5.109-48.215; P = 0.000), surgery (HR:1.493; 95% CI, 1.113-3.879; P = 0.036), and chemotherapy (HR:2.469; 95% CI, 2.279-7.361; P = 0.000) were also independent factors for OS in multivariate analysis. **Table 2** summarizes the outcomes of the univariate and multivariate analysis for DSS and OS. All of the independent factors were included in the predictive models for DSS and OS and visually presented as nomograms (**Figures 4A, B**). The calibration curves showed good consistency in the 3- and 5-year DSS and OS probabilities between the actual observations and the nomogram predictions in the training cohort (**Figures 4C, D**) and in the valiation cohort (**Figures 4E, F**). The C-index of the two nomograms for DSS and OS in the training cohort were 0.834 (95% CI, 0.773–0.894) and 0.829 (95% CI, 0.775–0.883), reflecting the good discrimination ability of the models. In the validation cohort, the C-index for the constructed nomogram to predict DSS and OS were 0.780 (95% CI, 0.670–0.891) and 0.769 (95% CI, 0.707–0.831).

**Table 2:** Univariate and multivariate analysis for disease specific survival and overall survival with training cohort.

Variables	UVA (DSS)			MVA (DSS)			UVA (OS)			MVA (OS)		
	HR	95% CI	*P*	HR	95% CI	*P*	HR	95% CI	*P*	HR	95% CI	*P*
Year of diagnosis	0.528	(0.364-1.115)	0.156				0.985	(0.897-1.043)	0.574			
Age at diagnosis	1.013	(0.975-1.011)	0.673				1.083	(1.114-1.538)	0.001	1.106	(0.847-1.178)	0.125
Race			0.640						0.328			
White	Ref						Ref					
Black	1.163	(0.651-2.486)	0.518				0.832	(0.548-1.379)	0.388			
Other	1.879	(0.214-3.981)	0.968				1.015	(0.164-2.642)	0.591			
Differentiation			0.081			0.325			0.026			0.207
Grade 1	Ref			Ref			Ref			Ref		
Grade 2	2.127	(1.003-4.921)	0.049	1.927	(0.683-4.538)	0.219	2.717	(0.183-5.467)	0.793	1.015	(0.245-2.739)	0.889
Grade 3	4.645	(1.682-10.682)	0.037	2.645	(0.908-5.682)	0.059	2.645	(0.704-5.468)	0.252	2.210	(0.579-6.427)	0.428
Unknown	3.495	(0.452-7.718)	0.421	2.016	(0.561-4.438)	0.382	4.329	(0.658-9.356)	0.443	3.170	(0.473-8.591)	0.657
ER/PR status			0.301						0.312			
ER-/PR-	Ref						Ref					
ER+/PR-	1.128	(0.613-2.233)	0.687				1.645	(0.438-4.414)	0.425			
ER-/PR+	0.818	(0.121-2.272)	0.385				2.190	(0.631-4.952)	0.532			
ER+/PR+	1.433	(0.298-3.512)	0.951				0.636	(0.246-1.479)	0.105			
Unknown ER or PR status	0.459	(0.181-1.136)	0.101				1.242	(0.609-2.072)	0.482			
Laterality			0.131						0.106			
Left	Ref						Ref					
Right	1.158	(0.665-1.643)	0.788				1.109	(0.873-1.732)	0.432			
Bilateral	5.288	(1.019-10.768)	0.063				5.267	(1.443-11.365)	0.003			
Tumor Location			0.000			0.078			0.001			0.318
Medial or Central	Ref			Ref			Ref			Ref		
Outer	1.358	(0.561-3.889)	0.282	1.649	(0.467-4.583)	0.453	1.485	(0.796-3.328)	0.172	1.698	(0.739-3.191)	0.326
Other	3.126	(1.009-8.793)	0.046	1.749	(0.577-5.756)	0.310	1.892	(0.980-4.478)	0.056	1.602	(0.669-3.896)	0.293
Unknown	6.388	(2.313-13.528)	0.021	3.679	(1.001-9.773)	0.050	3.365	(1.968-8.341)	0.001	1.769	(0.863-5.347)	0.134
Stage			0.000			0.000			0.000			0.000
I	Ref			Ref			Ref			Ref		
II	5.768	(1.412-11.826)	0.029	7.632	(1.105-16.439)	0.014	1.687	(1.056-3.653)	0.038	2.896	(1.061-6.386)	0.019
III	18.394	(3.675-45.852)	0.000	26.314	(2.476-68.421)	0.000	3.416	(1.786-8.816)	0.003	7.472	(2.659-14.351)	0.000
IV	35.235	(8.287-109.257)	0.000	55.968	(6.678-126.573)	0.000	11.615	(6.296-24.663)	0.000	21.876	(5.109-48.215)	0.000
Surgery												
Yes	Ref			Ref			Ref			Ref		
No	4.732	(1.879-10.856)	0.000	2.108	(1.063-3.585)	0.046	4.318	(3.013-6.463)	0.000	1.493	(1.113-3.879)	0.036
Radiation therapy												
Yes	Ref			Ref			Ref			Ref		
No	2.496	(1.103-3.467)	0.038	0.687	(0.365-2.014)	0.558	1.921	(1.421-2.926)	0.003	1.493	(0.687-2.396)	0.732
Chemotherapy												
Yes	Ref			Ref			Ref			Ref		
No/Unknown	0.694	(0.511-1.384)	0.259	3.543	(1.564-6.593)	0.003	1.102	(0.867-1.782)	0.203	2.469	(2.279-7.361)	0.000

CI, confidence interval; DSS, disease specific survival; HR, hazard ratio; MVA, multivariate analysis; OS, overall survival; UVA, univariate analysis.

**Figure 5:**
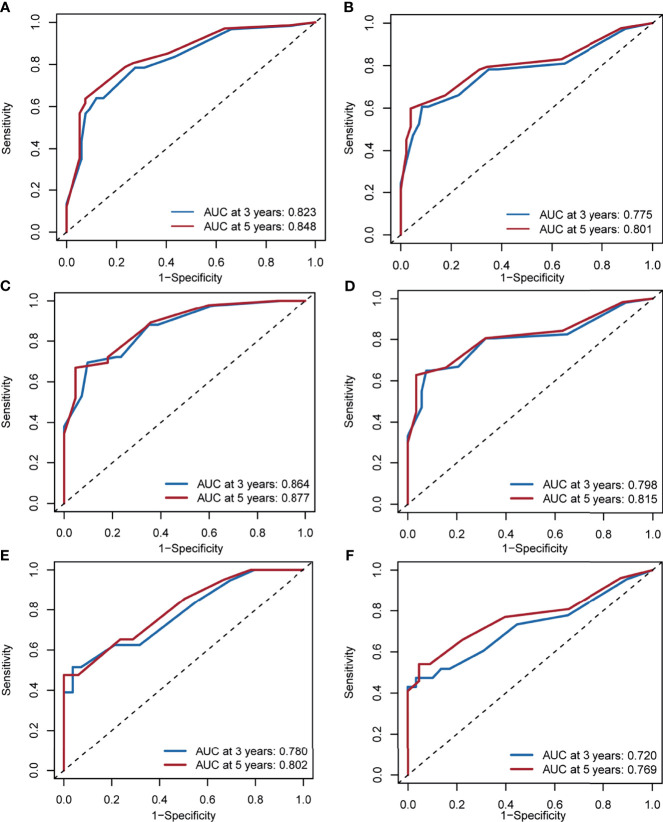
Area under the receiver operating characteristic curve for 3- and 5-year disease-specific survival and overall survival in the whole cohorts **(A, B)**, training cohorts **(C, D)** and validation cohorts **(E, F)**.

The AUCs for 3- and 5-year DSS in the whole cohort were 0.823 (95% CI, 0.753–0.879) and 0.848 (95% CI, 0.807–0.916), respectively (**Figure 5A**). The AUCs for 3- and 5-year OS in the whole cohort were 0.775 (95% CI, 0.711–0.839) and 0.801 (95% CI, 0.753–0.851), respectively (**Figure 5B**). In the training cohort, the AUCs for 3- and 5-year DSS were 0.864 (95% CI, 0.782–0.917) and 0.877 (95% CI, 0.821–0.938), respectively (**Figure 5C**). The AUCs for 3- and 5-year OS in the training cohort were 0.798 (95% CI, 0.723–0.872) and 0.815 (95% CI, 0.756–0.892), respectively (**Figure 5D**). In the validation cohort, the AUCs for 3- and 5-year DSS were 0.780 (95% CI, 0.637–0.878) and 0.802 (95% CI, 0.707–0.912), respectively (**Figure 5E**). The AUCs for 3- and 5-year OS in the validation cohort were 0.720 (95% CI, 0.597–0.844) and 0.769 (95% CI, 0.659–0.881), respectively (**Figure 5F**).

### Immunohistochemistry

**Figure 6:**
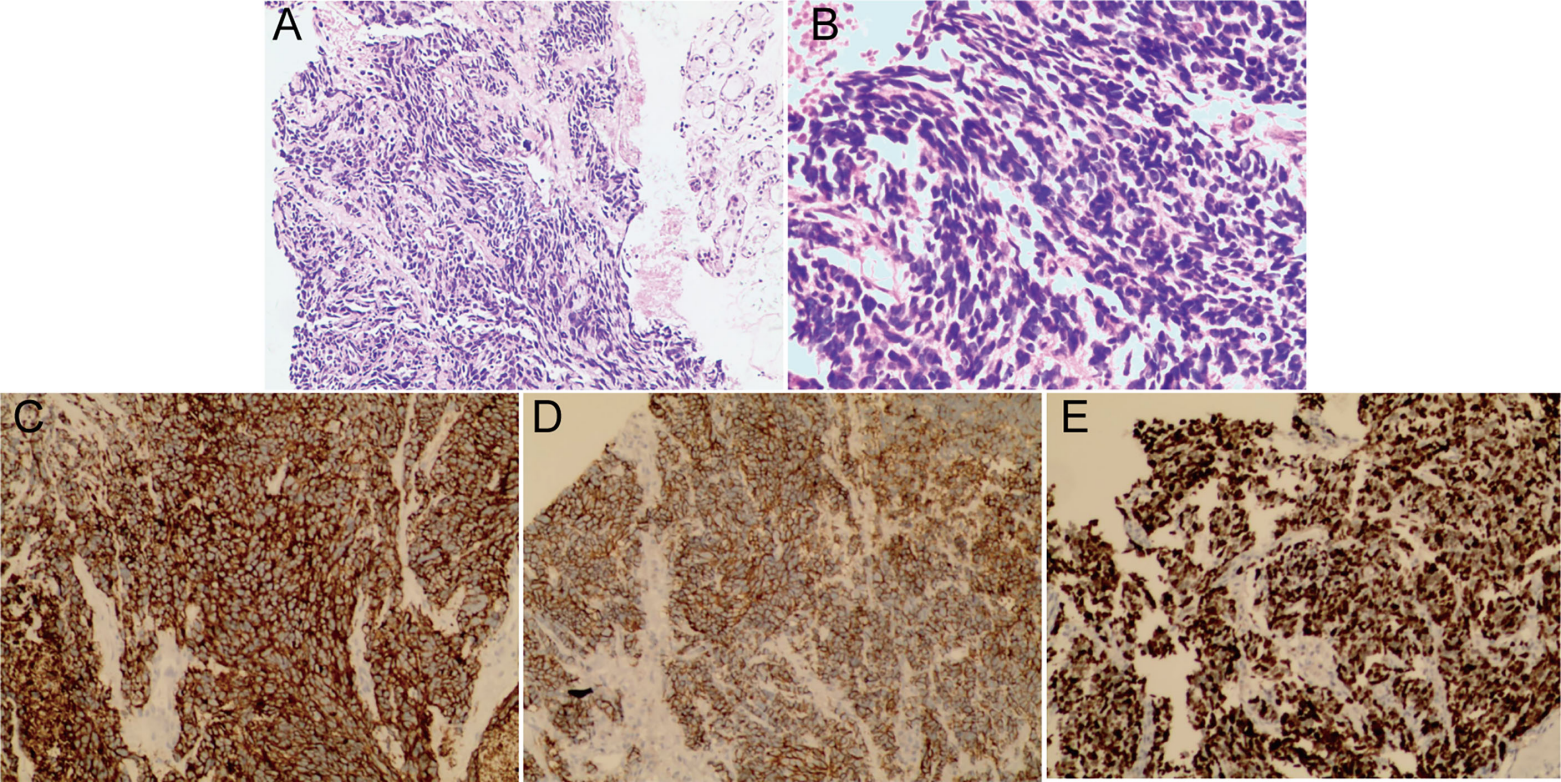
Histopathological examination of the stained specimen under a high-power field showed neoplastic cells arranged in solid sheets and short fusiform with light nuclei, fine chromatin, unclear nucleoli and a high mitotic rate consistent with the diagnosis of SCBC. (**A** 10x, **B** 20x). Immunohistochemical analysis showed that Syn **(C)** and CD56 **(D)** were positive and Ki67 was 65% **(E)**.

Histopathological examination of the stained specimen under a high-power field (10x, 20x) showed neoplastic cells arranged in solid sheets and short fusiform with light nuclei, fine chromatin, unclear nucleoli and a high mitotic rate, which is compatible with a SCBC diagnosis (**Figures 6A, B**). Immunohistochemical analysis showed that Syn (**Figure 6C**) and CD56 (**Figure 6D**) were positive and Ki67 was 65% (**Figure 6E**).

## Discussion

Primary SCBC is a rare malignancy, and the available information is limited to individual case reports or small case series in the literature. The clinical characteristics of 56 SCBC patients were analyzed in the study of Boutrid et al., but further survival analysis was not conducted (13). Similar outcomes were reported by Shin et al. (9) and Kanat et al. (14), who managed 9 and 7 patients, respectively. In this study, 323 SCBS patients between 1975 and 2018 were recruited through the SEER program. Our study is the largest scale report on SCBC with long-term follow-up and comprehensively analyze the incidence, survival, and prognostic factors of this malignancy.

SCBC is a rare malignant tumor, according to the outcome of our study, with a total incidence of 0.14 instances per million women each year between 1990 and 2018. The incidence reached the peak in 2003, and then a slight decline in SCBC was observed. However, the incidence of SCBC remained stable during the study period. Improved histological classification may serve as an influencing factor accounting for this phenomenon. Patients in the 65–69 age group had the highest incidence of SCBC, and the disease was more common in older women aged > 60 years, which is in line with a previous report (15). In terms of the prognosis of SCBC, the 5-year DSS and OS were 61.6% and 53.1%, respectively, and patients with SCBC had poorer survival than patients with any no uncommon type of invasive ductal carcinoma, who had 5-year DSS and OS rates of 89.2% and 83.2%, respectively. Patients with neuroendocrine neoplasms of the breast (containing 28.3% SCBC patients) had slightly higher 5-year DSS (63.4%) and OS (55.7%) rates (5). Stage-stratified prognostic analysis showed that patients with an initial diagnosis of stage IV disease had the worst DSS and OS. A high risk of recurrence and metastasis may contribute to poor survival.

Primary SCBC has similar biological and clinical characteristics to small SCLC. Most of patents with these two diseases have positive chromogranin A and synaptophysin in terms of Immunohistochemistry, but it is not necessary for diagnosis. While ductal carcinoma *in situ* promotes the diagnosis of breast carcinoma (16). More than half of the two malignancy patients have positive expression of TTF-1, which needs imaging methods to differentiate at diagnosis (13). In the early stages of the two diseases, SCBC has a more favorable outcomes compared with SCLC. And SCBC even has the best prognosis among the extrapulmonary small cell carcinomas, which attributed to the early detection and diagnosis of SCBC (2, 17).

Given the rarity of primary SCBC and limited reports in the previous literature, the optimum therapeutic modalities for this malignancy are still unknown. Surgery remains the major treatment for SCBC, including modified radical mastectomy and lumpectomy, especially for patients with disease in early stages of the condition. In this current study, 73.7% (238/323) of SCBC patients and 90.1% (9/91) SCBC patients with stage I or II disease underwent surgery. However, large cases and long follow-up studies comparing the outcomes of different surgical treatments are lacking. In addition to surgery, chemotherapy has a significant impact on the survival of SCBC, as shown in our study. Although a previous study showed that neuroendocrine neoplasms of the breast, gastrointestinal and pulmonary neuroendocrine system were not sensitive to chemotherapy, several case review series observed the prognosis benefit of adjuvant chemotherapy in the treatment of SCBC, especially in those with a high risk of recurrence (9, 13, 18). The mainstay chemotherapy regimens include anthracycline- and taxane-based chemotherapy regimens are commonly utilized for invasive breast cancers, whereas platinum-based chemotherapy is uregularly employed for small cell lung cancer (10, 14, 16, 19). Yildirim et al. proposed that platinum compounds and etoposide should be recommended for SCBC with Ki67 >15%; otherwise, an adriamycin-based regimen is preferred (20). The limited number of SCBC patients who received neoadjuvant chemotherapy with various chemotherapy regimens did not show satisfactory outcomes (9, 21, 22). With the advent of the era of immunotherapy, chemotherapy combined with immune checkpoint inhibitors (ICIs) could improve the survival of extensive-stage SCLC and advanced triple negative breast cancer patients based on the outcomes of phase III study such as IMpower 133 and IMpassion 130 (23, 24). Given the similar histologic and morphologic between SCBC and SCLC and triple negative breast cancer accounting for more than half SCBC, we consider that SCBC patients may benefit from ICIs treatment.

The use of radiotherapy in the management of SCBC patients remains controversial. Grossman et al. discovered that radiation had a survival benefit of radiotherapy among patients with extrapulmonary small cell carcinomas, including SCBC (2). Hare et al. analyzed the survival value of adjuvant radiotherapy in patients with localized and regional SCBC (25). However, no significant improvement in OS was observed in the adjuvant radiation group in their investigation, which is similar to the outcomes of our survival analysis and those of another study by Abbasi et al. (26). Molecular subtype may affect the treatment response. We observed that most SCBCs are TNBC. A recent BEATRICE trial-based retrospective study demonstrated that postmastectomy radiation therapy did not improve survival in TNBC with N0 or N1 status (27). Moreover, postoperative radiation is always recommended for patients with high-risk pathological characteristics, and the real benefit of radiotherapy is underestimated. Therefore, patients may potentially benefit from postoperative radiotherapy, and a larger sample size study is warranted.

Currently, immunohistochemical staining remains the most commonly used tool at the molecular biology level for SCBC. A wide variety of ER/PR statuses were observed among previous case series reports, while negative HER-2 status was commonly reported (9, 13, 28, 29). A high proportion of TNBC in SCBC was observed in both our study and others’ (10). To explore potential treatment targets, the genomic landscape of SCBC and small cell lung cancer was compared by McCullar et al. with next-generation sequencing (11). PIK3CA mutations only occurred in SCBC, with a 33% mutation rate, and a high level of TOP2A expression (77%) in SCBC was observed in their study. Therefore, PIK3CA and TOP2A were considered possible targets for treatment. PIK3CA mutations that activate the phosphatidylinositol-3-kinase (PI3K) pathway have association with poor prognosis in breast cancer patients (30). The SOLAR-1 trial found that adding alpelisib, a PI3Kα-specific inhibitor, provided a progression-free survival benefit and OS improvement in PIK3CA-mutated, hormone receptor-positive, HER2-negative advanced breast cancer patients (31). Alteration of TOP2A was reported to be related with restricted responsiveness to anthracycline-based chemotherapy in breast cancer (32). Thus, PIK3CA-targeted immunochemotherapy may also serve as an optional treatment option for SCBC, and the detection of TOP2A status before chemotherapy is necessary.

Several flaws exist in this study. First, the SEER database was used in this study. Although our study has the largest sample size of SCBC patients, the number of cases is still small compared to that of other common histologies. Subgroup analysis and external validation of the predictive model could not be conducted. Second, information on several variables in the SEER database is incomplete, such as molecular subtype, staging, regimen of chemotherapy and radiotherapy dose, which may affect the accuracy of the predictive model. Third, due to the uniform diagnostic criteria of SCBC, inconsistent recognition and diagnosis may exist during the study period. Last, inherent biases were inevitable in this retrospective analysis.

## Conclusion

SCBC is a rare, aggressive tumor that needs uniform multimodality therapies. The incidence of this malignancy is stable. Surgery and chemotherapy still play important roles in the treatment of SCBC and serve as independent factors, in addition to staging. A nomogram for predicting the DSS and OS of patients with initially diagnosed SCBC was established using the three above mentioned factors. Future external validation is needed, and prospective clinical trials are warranted to explore better treatment strategies.

## Data Availability Statement

The raw data supporting the conclusions of this article will be made available by the authors, without undue reservation.

## Author Contributions

JZ drafted the primary manuscript. KG and SJ conceived and supervised this research and revised the manuscript. SJ and GW contributed to the design of this research. JZ, YZ, BY, QC, and JJ collected and analyzed data. YM contributed to statistical analysis. All authors contributed to the article and approved the submitted version.

## Funding

This study was sponsored by Wuxi science and education project (FZXK 004), Suzhou Science and Technology Project (SYS2018083), Gusu Health Talent Program (GSWS2020067), Wuxi Taihu Lake Talent Plan, Supports for Leading Talents in Medical and Health Profession.

## Conflict of Interest

The authors declare that the research was conducted in the absence of any commercial or financial relationships that could be construed as a potential conflict of interest.

## Publisher’s Note

All claims expressed in this article are solely those of the authors and do not necessarily represent those of their affiliated organizations, or those of the publisher, the editors and the reviewers. Any product that may be evaluated in this article, or claim that may be made by its manufacturer, is not guaranteed or endorsed by the publisher.
